# The Calcium Wave Model of the Perception-Action Cycle: Evidence from Semantic Relevance in Memory Experiments

**DOI:** 10.3389/fpsyg.2013.00252

**Published:** 2013-05-06

**Authors:** Alfredo Pereira, Rafael Peres dos Santos, Rafael Fernandes Barros

**Affiliations:** ^1^Department of Education, Biosciences Institute, São Paulo State UniversityBotucatu, São Paulo, Brazil

**Keywords:** learning, memory, consciousness, perception, action, repetition, relevance

## Abstract

We present a general model of brain function (the *calcium wave model*), distinguishing three processing modes in the perception-action cycle. The model provides an interpretation of the data from experiments on semantic memory conducted by the authors.

## Introduction

**Figure 1:**
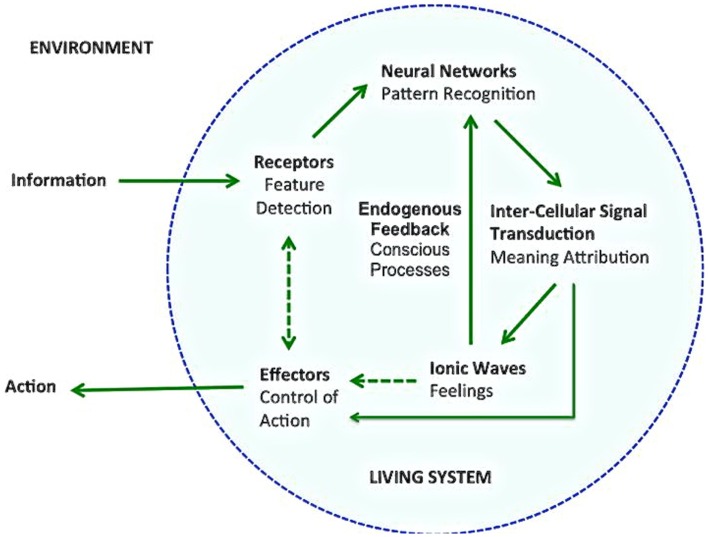
**A model of brain mental functioning in three modes**. In the first mode, external information is detected by specialized body/brain receptors, and directly influences effectors responsible for the control of action. In the second mode, there is a whole internal cycle of unconscious information processing, including pattern recognition in neural networks and (unconscious) meaning attribution, influencing the effectors. In the third mode, the attributed meaning triggers the formation of astroglial calcium waves, corresponding to the instantiation of feelings, which feed back positively or negatively on the pattern recognition process, forming an endogenous feedback cycle that also exerts an influence on the action effectors.

Brain information processing and the control of action can occur in three modes: automatic, unconscious, and conscious (Figure 1). Automatic and unconscious processes are often conflated, but several recent results about complex and flexible unconscious processing – not reviewed here – have contributed to disentangle them. The identification of mechanisms underlying the conscious mode has been a major challenge. What makes some mental processes conscious? The *calcium wave model* (Pereira and Furlan, 2010; Pereira, 2012) relates conscious action control with the presence of large ionic waves in astroglial networks of the brain, feeding back on the neuronal networks that prompt them.

In the model, dynamical information patterns are available in the environment of the conscious agent. They are received and processed, and the products can be used to guide action in the same environment. Linguistic, spoken, and/or written actions require a complex coordination of muscles that would not be possible without conscious processing (Morsella, 2005). The conscious mode requires, according to the model, the formation of an endogenous, positive or negative feedback that corresponds to current views of conjoint “bottom-up” and “top-down” activations, as in Adaptive Resonance Theory (Carpenter et al., 1992). The model relates such a “resonance” to reciprocal neuronal and astroglial network activations mediated by tripartite synapses in different intensities, corresponding to degrees of consciousness (Carrara-Augustenborg and Pereira, 2012).

## Evidence for a Role of Astroglial Calcium Waves in Conscious Processing

There is currently a good understanding of how astrocytes locally modulate neuronal function (De Pittà et al., 2011; Takata et al., 2011), reinforcing or depressing activity of post-synaptic neurons according to (still unknown) relevance filters. Pereira and Furlan (2010) have proposed a model of brain mental functions that relates large-scale calcium ion waves in the astroglial network with the “top-down” signal that modulates neuronal networks. Furthermore, these waves would correspond to the broadcasting of feelings about the content of the information processed by neurons. It is claimed that only with the generation of such feelings conscious processing occurs – otherwise (i.e., without feelings), the cognitive processing is unconscious. The relation of these waves with conscious processing is well documented by Thrane et al. (2012), showing that commonly used general anesthetics selectively suppress astrocyte calcium waves.

The generation of large-scale calcium waves begins with neuronal synchronization (Pereira and Furlan, 2009), producing a “carousel effect,” i.e., the neuronal induction of astroglial calcium movements (Pereira and Furlan, 2010; see also Ingber, 2012) simultaneously at many locations in the brain. The resulting calcium wave is both an integration of spatially distributed neuronal information and an affective reaction to the received information. This wave spreads in cortical tissue (Kuga et al., 2011; Navarrete et al., 2012) possibly by means of a “domino effect” (Pereira and Furlan, 2010), and feeds back on neurons, reinforcing or depressing their activity (as definitely proved by Han et al., 2013), probably according to the valence of the feeling (i.e., if the information content is experienced as being good, there is a positive feedback and neuronal activity is reinforced; and if it is experienced as being bad, there is a negative feedback and the activity is depressed).

Here we use this model to interpret empirical results on memory formation, the kind of results that have appeared in textbooks of cognitive psychology but have never been interpreted in light of a calcium wave model.

## Experiments

Learning can be reinforced by means of two factors: *repetition* of stimulation and *semantic relevance* of the stimulus. In cognitive neurobiology, these strategies correspond respectively to *temporal summation* of stimuli and *spatial summation* induced by the matching of bottom-up (sensory) and top-down (attentional/motivational) signals.

We executed a series of cognitive experiments addressing the possible roles of stimulus repetition and semantic relevance in the formation of short-term declarative (conscious) memory (see Marques et al., 2010; Barros et al., 2011). A population of 157 undergraduate students was presented with linguistic stimuli of two kinds: unrepeated sentences containing information relevant (e.g., about fellowships and sports) or not to their lives, and repeated sentences with irrelevant information only (e.g., about events in distant small towns). The relevance or irrelevance of the sentences for the target population was previously checked by means of piloting.

**Figure 2:**
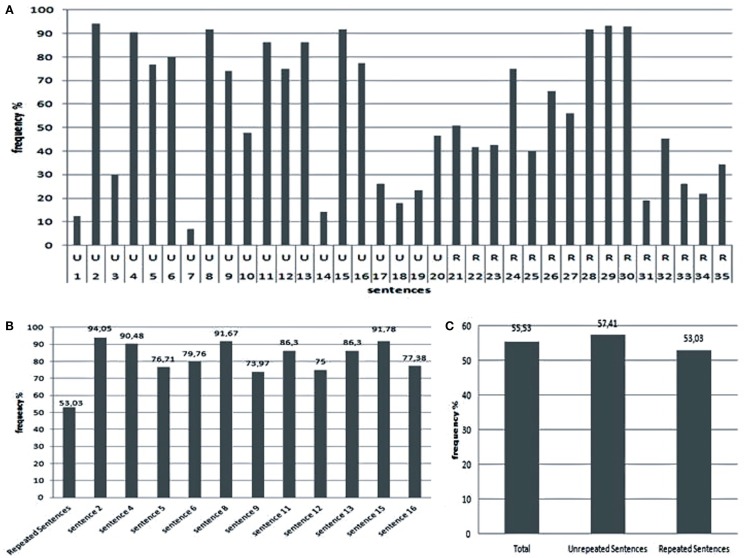
**Results of classroom experiments on declarative semantic memory formation: (A) frequency of correct answers for unrepeated relevant or irrelevant (U) and repeated irrelevant (R) sentences: (B) comparison of the average of correct answers for repeated irrelevant sentences (first column) with highly remembered single unrepeated relevant sentences; (C) comparison of average frequency of correct answers for repeated irrelevant against unrepeated relevant and irrelevant sentences**.

After a brief, sequential presentation of the sentences using a screen projector, the students were asked to answer a written questionnaire containing one question about each sentence. The results indicate a within-subjects effect: unrepeated relevant information was more efficient for semantic memory formation than repeated irrelevant information (Figure 2). Control unrepeated sentences conveying irrelevant information were poorly remembered.

## Discussion

The above results can be understood in terms of the calcium wave model of conscious action control (there are, of course, other models that would be consistent with the results). According to the model, our obtained results can be understood as an effect of astroglial modulation of neuronal activity: the triggering of a strong endogenous positive feedback by relevant information contents, but not by non-relevant ones (these would elicit a weaker positive feedback, or even a negative one).

In sum, comparing the effect of presentations of relevant-and-unrepeated against irrelevant-and-repeated sentences within-subjects, our model predicts a difference in degree and valence of calcium wave activation. On the one hand, single presentations of relevant sentences would elicit *a stronger, positively valued astrocyte calcium wave* that reinforces neuronal activity, leading to an increase of calcium ion entry in the post-synaptic neuron. These ions possibly bind to calmodulin and related kinase proteins, activating signaling pathways that support memory formation. On the other hand, repeated presentations of boring information would lead to *a weaker, negatively valued wave* that does not produce such a reinforcement, and in some cases possibly leads to an inhibition of the corresponding neuronal receptors by means of glial transmitters. The latter possibility would explain why some irrelevant sentences presented five times were less remembered than other irrelevant sentences presented three times (for details of experiments and statistical analysis of the results, see Marques et al., 2010 and Barros et al., 2011).

We hope that this non-mainstream model of consciousness, along with our presentation of the kind of data that could be used to support such a model, will spur new ways of theorizing about the challenging topic of consciousness and action control.

## Conflict of Interest Statement

The authors declare that the research was conducted in the absence of any commercial or financial relationships that could be construed as a potential conflict of interest.
